# The Recent Progress China Has Made in Green Mine Construction, Part I: Mining Groundwater Pollution and Sustainable Mining

**DOI:** 10.3390/ijerph19095673

**Published:** 2022-05-06

**Authors:** Shuai Li, Lifeng Yu, Wanjun Jiang, Haoxuan Yu, Xinmin Wang

**Affiliations:** 1 School of Resources and Safety Engineering, Central South University, Changsha 410083, China; shuaige@csu.edu.cn (S.L.); 19507486989@163.com (L.Y.); 8210183016@csu.edu.cn (X.W.); 2 China Road & Bridge Corporation, Beijing 100011, China; jiangwanjun777@gmail.com

**Keywords:** green mining, environment protection, sustainability

## Abstract

With the development of technology, the concepts of “green” and “sustainable” have gradually been popularized in all walks of life. With the continuous development of the world mining industry, the efficiency of resource development in various countries has been improved, but mining activities and production will undoubtedly bring many environmental pollution problems. As a mining power, China is one of the first countries to put forward the concept of “green mining”. Over the years, as people emphasize safety and environmental protection, green mining technology has become the hot topic. At the same time, groundwater pollution caused by mining has become the focus of China’s “green mine construction”: with the continuous development of mining, mining activities and production will also undoubtedly bring significant environmental pollution. The environmental pollution of the mined area has a vital influence on the surrounding environment. The pollutants mainly come from mining operations and production of the mineral processing industry, including process wastewater, gas waste, smelting slag, etc., which are all acidic. Acid mine drainage (AMD) occurs in the process of mining production, due to the structure of minerals and the complex reactions between oxygen and minerals, and results in heavy metal ions leaching into groundwater. Once the groundwater is polluted, it will slowly flow to the surrounding area, resulting in the migration and diffusion of pollutants in the groundwater, affecting the surrounding rivers, farmland, and drinking water for residents. In recent years, environmental damage caused by groundwater pollution from underground mines in Shijiazhuang, China, and Selangor, Malaysia, has had a negative impact on rivers, farmland, and human health. At the same time, the paper introduces many key technologies of green mine construction, such as the backfill mining method. In cooperation with China Road & Bridge Corporation, this paper also introduces the progress in the reuse of mining waste, especially the use of mining waste as aggregate to prepare concrete materials for road and bridge construction. This information article introduces the development status of green mine construction in China and briefly reviews the key technologies of green mine construction in China.

## 1. Introduction

With the development of science and technology, the concepts of “green” and “sustainability” have been gradually popularized in all walks of life.

In the area of tourism, Pan, S. Y. et al. [1] summarized the interrelationship between tourism and sustainability from an interdisciplinary perspective, reviewed the development of sustainable tourism and the concept of the green economy, and discussed the key interdisciplinary elements of sustainable tourism. In the area of transportation, Shah, K. J. et al. [2] proposed the concept of green or sustainable transport, in which the expansion of transport systems should be carefully planned for global sustainability. In the area of materials, energy, and industrial production, Kumar, A. et al. [3] and Pan, S. Y. et al. [4] put forward the concept of green materials, green energy, and green production, and they advocated the reuse of waste and the re-treatment of wastewater. In the area of agriculture, Chen, C. Y. et al. [5] believed that the use of unconventional water sources for irrigation is critical to ensuring water sustainability, and they wanted to implement innovative technologies to promote and facilitate the reuse of unconventional water, using treated wastewater for agricultural irrigation, which is called “sustainable irrigation for agriculture”. In the area of the chemical industry, Chen, B. C. et al. [6] developed a novel and sustainable method to adsorb and recover feedstock in the exhaust gas downstream of the distillation system and condensation system during the recovery process of mercury-containing equipment. In the area of urban construction, Sun, Y. et al. [7] put forward the concept of sponge city. A sponge city, as an advanced rainwater management technology, plays a crucial role in urban transformation and new construction. Meanwhile, they proposed that urban infrastructure should be gradually transformed into green infrastructure. In the area of the resource industry, especially mining, Yu, H. et al. [8] and Li, S. [9], as mining researchers in China, also introduced the construction and development of green mines in China.

For industrial production and resource extraction activities, “green” is undoubtedly one of the keywords in recent years, especially in the mining industry, because the exploitation of resources always inevitably causes many environmental pollution problems. Therefore, in 2019, Pan, S. Y. et al. [10] proposed that any process, product, or service that reduces negative environmental impacts while protecting human health and ecosystem quality, which coincides with the concept of a green mine in the Construction Code for Green Mines in Metal Mining Industry issued by the Ministry of Natural Resources of China [11] be categorized as sustainable. In China, a green mine [11] is defined as a mine that implements scientific and orderly mining in the whole process of mineral resources development, controls the disturbance of the ecological environment in the mining area and surrounding areas within the controllable range, and realizes an ecological environment, scientific mining methods, efficient resource utilization, digitalized management information, and harmonious mining community.

Of course, not only China but Finland [12] also put forward the concept of “green mining” in 2011. In addition, researchers from Australia, Ghana, Sweden, and the United States have carried out many relevant studies on green mining technology and management [13,14,15].

China is a mining power in resource exploitation, but there are serious problems of safety, hidden danger, environmental pollution, and resource waste in mining development; it is very important to promote green mining technology, build green mines, and realize the transformation and upgrading from extensive mining mode to green non-waste mining mode. Thus, this paper, an informative article, as a medium to lead readers to China’s green mining construction, mainly introduces two aspects:

(1) Mining pollution, especially groundwater pollution;
(2) Development Course of Green Mine Construction in China.

## 2. Mining Groundwater Pollution

China has achieved rapid economic growth over 30 years, especially in the mining industry (both coal and metal mines) [16,17].

While China is the world’s third-largest coal producer, rapid industrialization and urban expansion have now caused considerable land-use intensification [18,19,20,21]. Coal mining has caused great damage and pollution to the surrounding environments [22,23].

In terms of metal resources, the vast majority of gold, silver, iron, cobalt, nickel, copper, aluminum, and other metal resources in China come from the mining operations of metal mines [24]. At the same time, mineral resources development activities are accompanied by serious environmental pollution and ecological destruction, solid waste, liquid waste, gas waste, and other pollutants, as well as ground collapse, vegetation destruction, slope instability, and other environmental safety problems. These problems have become the universal impression of the mining industry by the global public [25]. At the same time, the environmental problems brought by mining are not only related to ecological security but also have a certain impact on the economy, community, and culture [26,27].

Therefore, in order to solve the above problems, the most fundamental way is to start with mining technology, realize the coordinated development of resource development and environmental protection, reduce the discharge of pollutants and damage to the environment, so as to eliminate or reduce the negative impact on the economy, society, and culture [28].

The main purpose of green mine construction is to eliminate or lessen the impact of mining activities and mining production on the environment. The main problem in mine production activities is the discharge of wastewater, which leads to environmental disasters such as mine environmental and groundwater pollution [29,30].

### 2.1. Chemical Pollution Caused by Mining Operations and Production

Groundwater pollution caused by acid mine drainage is undoubtedly a very important reason for the implementation of green mine construction [30,31].

The production of copper, iron, lead, zinc, and their alloys has grown significantly over the past decade, owing to surging demand from industries, such as construction and the automobile industry in recent years, which has led mining companies to invest heavily in mines. However, a large volume of water is needed in the mining process and the beneficiation process. Therefore, the whole production process of mines tends to pollute the environment of the mine. Chemical pollution occurs in the following ways:

(1) During the mining process, heavy metal ions leach into groundwater and as a result, acid mine drainage (AMD), caused by complex reactions between minerals and oxygen according to the structure of minerals, occurs [32].

Acid Mine drainage (AMD) during the mining process will increase the acidity of surrounding rivers and lakes, which has a great impact on the survival of aquatic organisms. In 2000, a study by Leblanc, M. et al. [33] found that the Tinto River region in southwestern Spain was heavily polluted by mining operations. The Tinto River which is called the “Red River” by locals has a pH of 1.5 to 2.5, is highly acidic and heavily contaminated with heavy metals. In 2019, Affandi, F. A. and Ishak, M. Y. [34] discussed the risks of acid and heavy metal pollution caused by mining activities to fish and fish population decline, and believed that metal pollution and bioaccumulation caused by mining activities were the biggest threats to fish survival.

In addition, the pollution caused by mining operations also has a great impact on many other factors, such as groundwater. As China has significant groundwater agricultural irrigation, mine pollution will undoubtedly have a great impact on farmland and crops. Due to China having long realized that groundwater pollution has a negative impact on agriculture, Yang, J. et al. [35] established the entropy cloud model of heavy metals as early as 2016 to evaluate farmland soil pollution around mining areas. In 2018, drawing on previous research, Kuang, Y. et al. [36] then focused on the study of the impact of mining activities in China on mercury (Hg) concentrations in farmland soils, and later in 2019, Shen, Z. et al. [37] did the same research with Kuang, Y. et al. [36], within China. Interestingly, Liao, J. et al. [38] conducted an overall study on the impact of acid mine drainage (AMD) on the surrounding farmland and crops from the perspective of ecotoxicology and environmental safety in 2016.

Although in 2021, Shuai Li et al. [23] proposed that alkaline filling materials can be used to fill out-mined areas of underground mines in order to solve the problem of AMD pollution, the most effective solution to mine pollution at the present stage is still prevention, which means that pollutants need to be controlled at the mining stage rather than treated later [39].

(2) During the beneficiation process, the concentrator mainly uses a large volume of water and chemicals to leach the ore. This is followed by flotation, and then the discharge of wastewater. However, if the wastewater treatment is not appropriate, it will lead to the pollution of the environment and groundwater.

In addition, sulfur pollutants such as carbon monosulfide (CS) and other pollutants will also be produced, mainly in the environment around the industrial site of the concentrator. For example, Zhang, C. et al. [40] proposed that a large amount of zinc neutral leaching residue (ZNLR) containing cadmium was continuously produced in the electrolytic beneficiation of zinc ore, which would cause serious cadmium pollution. In order to solve this problem, he developed a new process to reduce zinc leaching by using sulfur dioxide (SO_2_) as a reducing agent, which solved the problem of mineral processing with the cadmium pollution factor. In 2021, Chinese researchers Tian, J. et al. [41] put forward a new process for the comprehensive utilization and safe disposal of arsenic and alkali slag combining beneficiation and metallurgy, in order to prevent and control arsenic pollution in the beneficiation process.

Outside China, water pollution caused by mineral processing has become a concern of experts in recent years. In 2015, Motaung, S. R. et al. [42], researchers from South Africa, proposed that waste recovery and comprehensive utilization should be carried out in the process of beneficiation drainage and AMD so as to promote the recovery of treatment costs and prevent environmental pollution caused by gypsum waste dumping. In 2021, Malek, A.; Rao, G. R. and Thomas, T. [43], researchers from India, proposed that it is essential to remove heavy metals from wastewater before the discharge of beneficiation wastewater. Since the wastewater treatment process requires significant energy, they also proposed using clean energy in the wastewater treatment process.

**Figure 1:**
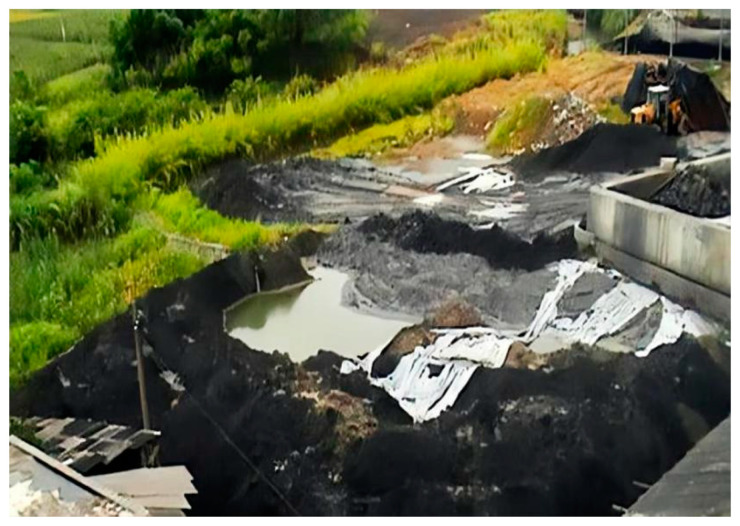
(Source from the Internet: https://www.163.com/news/article/A0L5TE8900014AEF.html, accessed on 26 March 2022) The discharge location of the He-chi Xin-cun Concentrator from China [44].

The discharge location of beneficiation wastewater and waste slag of the concentrator also has a great impact on environmental pollution. Some concentrators in China, such as the He-chi Xin-cun Concentrator [44], have discharged waste slag and wastewater near farmlands and the river, as shown in Figure 1 (Source from the Internet: https://www.163.com/news/article/A0L5TE8900014AEF.html, accessed on 26 March 2022). Pollutants can seep into farmlands and the river either through migration or through groundwater flow, which is very harmful to residents.

Although China has a large land area, its population is relatively large. Therefore, there are residents living near mines in China. Therefore, the discharge of wastewater and waste from mines has a great impact on the lives of residents, especially in rural areas of China, where residents obtain drinking water from wells. Once groundwater is polluted by mining activities and production, the health of residents will be harmed.

(3) At the same time, the additives used in the mining activities might also cause potential contamination. Spills during transportation or storage lead to contamination beyond reasonable limits from other sources, including soil and ground water, and thus pose a continuing threat to human health.

Backfill is a very important part in mining operations; backfill in mining operations refers to using some materials to fill the gap of the mined part inside the mine fully to avoid the occurrence of landslides, mine collapse, and other accidents. However, in order to improve the properties of backfill materials, many chemical additives are often needed, which may cause potential pollution, as the use of significant water, cement, other cementing materials, and some chemical additives to produce filling materials occurs. When the filling slurry enters the mine, it will undoubtedly fully contact the mine environment, causing groundwater pollution.

Saedi, A.; Jamshidi-Zanjani, A. and Darban, A. K. [45,46] have undertaken many studies on additives of mine backfill materials in the past decade. Meanwhile, they also focused on studying the environmental impact of chemical additives in mine backfill materials. In 2021, they undertook a comprehensive review [46] on the environmental impact of chemical additives in mining backfill materials and proposed that although the chemical additives can improve the performance of backfill materials, the chemical additives will undoubtedly have a serious impact on the mine environment. Therefore, they hoped that there will be an “environmentally friendly” chemical additive in the future for the mining backfill, which can create better conditions for mine environmental protection.

Therefore, the Chinese government requires mining enterprises in China to control the disturbance of the ecological environment in mining areas and surrounding areas within a controllable range during the whole process of mineral resources development.

### 2.2. Mine Groundwater Quality Modeling

A number of researchers from different countries have tried to use water quality modeling or water pollution prediction models to assess the environmental impacts of industrial or mining activities. They have endeavored to highlight the necessity for green mine construction by qualitative analysis of the extent of mining pollution.

To some extent, a water quality model is an important evaluation index and forecasting tool for water pollution, and is important for people’s lives. In 2020, Izni Zahidi et al. [47] established the water quality model for river activity management in Malaysia. It is an important research which revealed the trends in pollutants in this area over the years and the percentage of time each pollutant exceeded a certain category. Meanwhile, the author also undertook the same research on mining water pollution. For groundwater, the establishment of a water quality model also has the same effect as the rivers’, especially in China where there is significant groundwater agricultural irrigation. Therefore, the quality of groundwater has a great impact on agriculture.

In 2019, Su, F.; Wu, J. and He, S. [48] applied the Set Pair Analysis-Markov Chain model to evaluate groundwater quality. Their study took groundwater quality monitoring data from 1996 to 2015 as an example and used the Set Pair Analysis-Markov Chain model to predict groundwater quality. Based on the analysis of groundwater quality sample data over an extensive time span, this modeling method can evaluate and predict the change process and trend of groundwater quality, which is more suitable for urban groundwater quality evaluation and prediction. They used this modeling method to evaluate and predict the groundwater quality of Xi’an City, China, which has promoted the development of Xi’an city and the reform of environmental policy.

However, prior to this in 2016, Vadiati, M. et al. [49] developed a decision-making method based on Mamdani fuzzy logic to evaluate groundwater quality according to relevant indicators. Focusing on exploring the impact of groundwater quality on drinking water, they developed the Mamdani fuzzy reasoning model using widely accepted groundwater quality indices: Groundwater Quality Index (GQI), Water Quality Index (WQI), and Groundwater Quality Index (GWQI). Notably, they assessed the drinking water quality of 49 samples collected seasonally from groundwater resources in Iran’s Sarab Plain from 2013 to 2014, and they found that the Mamdani fuzzy reasoning model is reliable and flexible in the assessment of drinking water quality. Their study has developed a new method for modeling groundwater water quality modeling and evaluation. Obviously, they use a reasonable method to model groundwater water quality and consider various water quality impacts on pollutants to assess the water quality of the drinking water from groundwater, which has played a guiding and enlightening role for some researchers studying groundwater quality modeling methods in heavy metal polluted areas.

For example in 2019, Gad, M. and El-Hattab, M. [50] drew on previous research and used the Heavy Metal Pollution Index (HPI), Heavy Metal Evaluation Index (HEI), Pollution Index (PI), and DRASTIC model to assess the groundwater quality of El Fayoum Depression in the western Desert of Egypt. They took samples through groundwater logging, measuring pH, TDS, EC, Al, Ba, Cd, Cr, Cu, Fe, Pb, Mn, Ni, Sb, and Se using standard analytical methods. It is worth mentioning that this is a very standard heavy metal analysis method of environmental pollution elements. In addition, they used the 3D Pie model to visually show the relative pollution index of various heavy metals in the area. Although Gad, M. and El-Hattab, M. [50] have undertaken these valuable studies, the purpose of their research was initially only to verify the applicability of the integration of water pollution indices and the DRASTIC model in assessing groundwater quality. However, there is no doubt that their research provides significant ideas for engineering researchers to detect environmental pollution, especially researchers of major in mining and environmental engineering.

In 2021, Mal, U. and Adhikari, K. [51] introduced a new method that attempts to classify mine groundwater according to pollution level and then allocate it to specific uses, such as drinking water and irrigation. They grouped groundwater by its physical and chemical parameters according to its toxicity and used the Analytic Hierarchy Process (AHP) to assign weight to each group to determine where each part of the groundwater went. This is the most advanced research on underground mine water quality modeling, and their model establishment of the WQI is based on the relative weight of each parameter of the Drinking Water Quality Index (DWQI) and Irrigation Water Quality Index (IWQI) allocated by the AHP.

In addition, the research by Mal, U. and Adhikari, K. [51] has attracted significant attention, because they not only built a more perfect mining area groundwater quality evaluation model, but, more importantly, put focus on agriculture.

For farmland, the change in ions in groundwater has a great influence on the growth of crops. If calcium ions are absent from groundwater, soil permeability decreases. At the same time, calcium and magnesium are generally in balance in groundwater, however, a higher concentration of magnesium in the water can increase soil alkalinity and reduce the ability of crops to yield [52]. They analyzed the Lower Gondwana mine groundwater in India, mainly ion change before and after the monsoon, which is represented by a box diagram.

### 2.3. Influence of Groundwater Pollution on Farmland, Crops, and Human Health in Mining Areas and Industrial Sites

It can be seen from the research of Todd, D. K. et al. [52] that if mine pollution causes an ion imbalance in farmland soil, which leads to a reduction in crops, it will cause serious harm to agriculture. Groundwater pollution has been increasing in both urban and rural areas in the past 20 years. Groundwater pollution has a number of adverse effects on agricultural production, especially crop yields.

First, for farmland irrigated with alkaline well water over a long time, the soil structure changes to a certain extent over time, and the soil becomes rigid, which affects the normal cultivation of farmland. If nitrate levels are high in irrigated water, the overall immunity of crops and their ability to resist pests and diseases is gradually reduced. For food crops, if the crops contain excessive nitrate, the protein content in food gradually decreases. This is one reason why the nutritional value of food crops decreases, which reduces the function of food crops themselves. For vegetable crops, if they are irrigated by polluted groundwater for a long time, they are prone to decay, which makes them unable to be stored and transported normally, resulting in the loss of nutritional value of vegetables, which has an impact on people’s health.

At the same time, if the content of sulfate and chloride ions in the well water exceeds the standard, the normal growth of crops is affected to varying degrees, resulting in a large area of crop yield reduction, and is also the main reason for a sharp decline in the overall quality of crops. In a word, on the basis of the development and utilization of groundwater resources, it is necessary to effectively protect groundwater resources and the ecological environment on which people rely for survival. Otherwise, groundwater resources will be polluted and irreparable economic losses will eventually be caused.

For about 70 years, mining activities have largely led to the pollution of groundwater resources. Farmland near mining areas or industrial areas, polluted by heavy metals from decades of mining activities, often becomes barren land or produces inferior crops over time.

Since 2010, groundwater pollution from the Tonglushan mine in Daye City, China, has become increasingly serious, disturbing the already balanced earth surface system, damaging local ecosystems, and reducing the natural production capacity, especially the output value of crops.

Researchers Zhang Li and Wan Taiping [53] from China collected 17 samples of rice and farmland soil, respectively, in the Tonglushan mining area of Daye City in 2012, and analyzed the Cu, Pb, Zn, Fe, Mn, Ca, and Ni contained in them. They found that, by comparing the background values of the soil in this area, all the elements in the known background exceeded the background values, and the range of exceedance was large, up to 43 times, with the minimum being 1–3 times, indicating that Cu, Pb, and Zn in the soil in this area had been polluted in slightly different pollution degrees. Their research revealed that the pollution of underground water from mines is a very serious cause of damage to farmland soil. Furthermore, in China, Li, K. et al. [54] studied the influence of heavy metal pollution in a coal mining area in Henan, China, on the surrounding farmland soil in 2018, and conducted a comprehensive assessment of heavy metal pollution in the soil and waste dump. What was interesting about their study is that, in addition to measuring the concentrations of cadmium (Cd), lead (Pb), copper (Cu), zinc (Zn), and chromium (Cr), they also predicted the spatial distribution of toxic metals and pollution sources and studied the potential ecological risks and potential health risks. Moreover, in 2018, China, Li, P. et al. [55] believed that groundwater was crucial to the sustainable development of the Loess Plateau, but due to natural factors and man-made pollution, groundwater quality in this region was generally poor. Therefore, they investigated the suitability of groundwater for domestic and agricultural uses in Yan ‘an city on the Loess Plateau in China and assessed its impact on sustainable groundwater management in the plateau. Their investigation showed that mining pollutants were the most serious for groundwater pollution. Sadly, residents in Loess Plateau have historically used groundwater to irrigate farmland, which now seriously affects the growth of crops and the health of residents. Recently, Anh T P Hoang, Prinpreecha, N. and Kim, K. [56] found that elevated arsenic levels in rice from mining areas in India, Bangladesh, and Vietnam may have come from arsenic-contaminated groundwater, and they suggested that arsenic contamination in groundwater was most likely due to mining activities. Their study showed that mining activities and associated residual wastes significantly influence arsenic contamination of food crops, as rice samples from these areas were highly contaminated and the highest total arsenic concentrations recorded were 3–4 times higher than the maximum levels proposed by the Codex Alimentarius Commission.

All of these above studies have shown that groundwater pollution from mining operations and production is very harmful to the surrounding agricultural development and the health of the residents nearby, and that pollution spreads from the center of mining operations and production with a downward trend.

## 3. Development Course of Green Mine Construction in China

The idea of sustainable development has extremely rich connotations, involving many aspects of nature, environment, society, economy, science and technology, and politics. Obviously, green mining is also an important part of it [57]. In the field of mineral resources exploitation and utilization, it refers to the combination of ecological environmental protection and economic benefit development as an organic whole. Economic development should take into account the current carrying capacity of the natural ecological environment, so that the environment and resources can not only meet the needs of economic development, but also meet the needs of human long-term survival and development. Therefore, in the field of mineral resources development, green mining is one of the technical means of sustainable development, has a very important position [58,59].

Specifically for China, the definition of green mining can be further divided into [60]:

(1) Adopt advanced mining technology and mechanized mining equipment to realize the safe and efficient recovery of mineral resources and prevent the subsidence and collapse of goaf and surface;
(2) Resource utilization and harmless discharge of solid waste generated by mines to protect the ecological environment on the surface;
(3) To achieve recycling of mineral processing tail water or standard discharge.

### 3.1. The Development Course of Green Mining in China

Although China is a mining power, the development of China’s mining industry started late. Therefore, in the early exploration process of green mining, China largely borrowed from the development process of green mining in western countries and experienced the following three stages:

(1) The first stage: Green mining areas stage (before 1945).

China drew lessons from the concepts of “sustainable mining” and “environmental protection in mining areas” proposed by western countries such as Britain and the United States and focused on vegetation protection and greening in mining areas.

**Figure 2:**
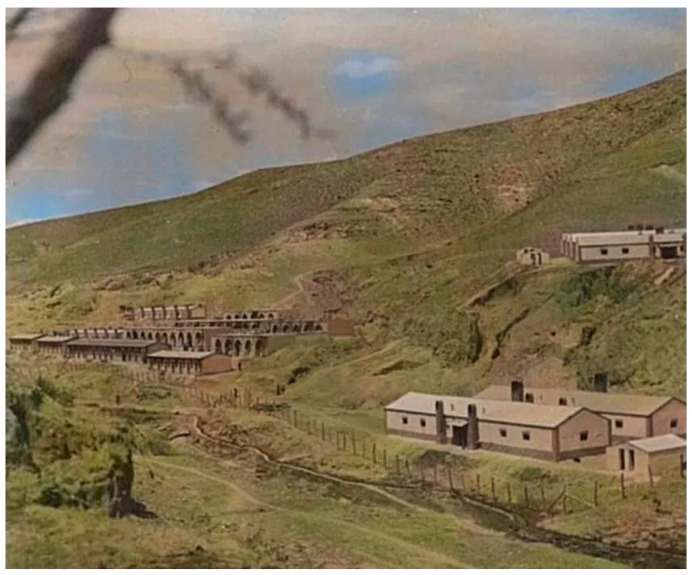
(Source from the Internet: https://www.sohu.com/a/317121804_166939, accessed on 26 March 2022) China’s earliest green mine: Shanxi Meiyukou Coal mine [61].

Especially in Shanxi during World War II, China not only focused on coal mining, but also integrated the mining industry with agriculture and animal husbandry, developing resources while protecting the environment and developing one of China’s earliest green mines, as shown in Figure 2 (Source from the Internet: https://www.sohu.com/a/317121804_166939, accessed on 26 March 2022), according to Chinese media reports [61].

(2) The second stage: Comprehensive utilization of resources stage (1945 to 2000).

After the end of World War II in 1945, the global economy developed rapidly, and human society’s consumption of natural resources increased at an unprecedented speed. More and more scholars realized the preciousness and scarcity of the Earth’s resources and put forward proposals to improve the comprehensive utilization rate of mineral resources and reduce the loss and waste of resources. At this time, the concept of green mining was extended from simple mining greening to comprehensive utilization of resources.

Increasingly serious environmental problems due to extensive mining, such as Shaanxi Shenmu county coal mining by mining in farmland were persecuted [62], and through the county Hunan province, the deadly residents health hazard caused by tailings pollution [63]. At this stage, China gradually realized that the problem, and there was a need to strengthen the integrated application of resources and comprehensive waste treatment and it became a primary target of China during this period.

(3) The third stage: Green and intelligent mining stage (2000 to present).

**Figure 3:**
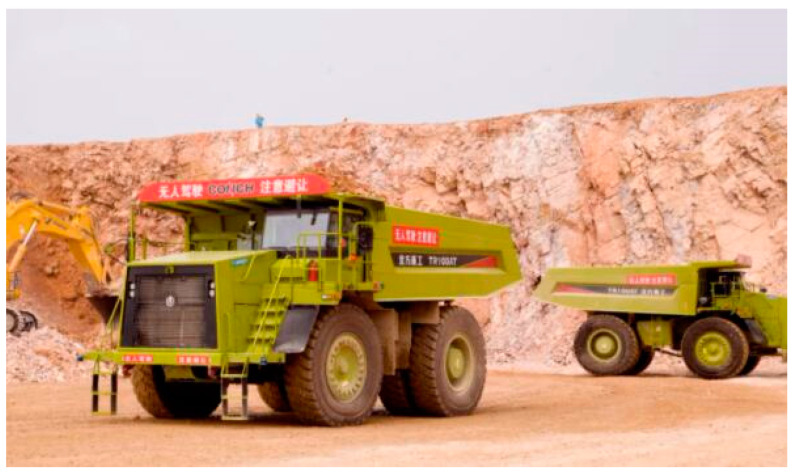
(Source from the Internet: https://www.xianjichina.com/special/detail_482389.html, accessed on 26 March 2022) China Conch Group’s driverless mining trucks [64].

After entering the 21st century, resource shortages and environmental pollution became the common problems restricting the development of all countries in the world. Keywords such as “green”, “sustainable”, “responsible”, and “transparent” have gradually become the basic concepts of global mining development, and the concept of green mining has gradually become more comprehensive, clear, and in line with the actual situation. Green mining technology has improved and been rapidly promoted and applied. For example, according to Chinese media reports, China Conch Group [64] (as shown in Figure 3) has combined the digitization of mine information with intelligent production factors to truly achieve green mining.

**Figure 4:**
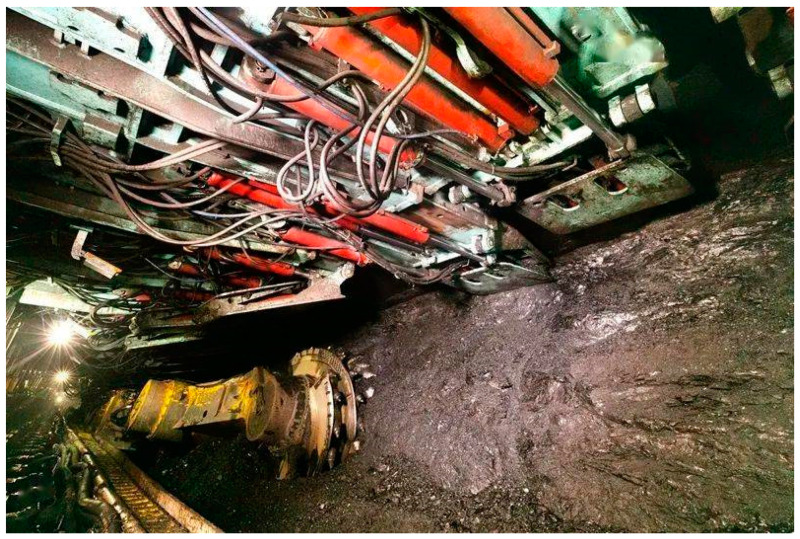
(Source from the Internet: https://www.sohu.com/a/433532471_120207614, accessed on 26 March 2022) China’s earliest green mine: Shanxi Meiyukou Coal mine [65].

In 2020, China Huaibei Mining Group [65] proposed to fully advance into the new era of intelligent mining, which adhered to the development of “green mining”, “intelligent mining”. At present, Huaibei Mining Group has made great achievements in intelligent coal mining, as shown in Figure 4, which shows the intelligent fully mechanized mining technology and equipment in coal mining developed by Huaibei Mining Group.

The development of intelligent mining technology also plays a vital role in the construction of green mines. Yu, H. et al. [66,67,68] have endeavored to build an intelligent integrated underground ventilation and transportation system since the end of 2020. Li, S. [69,70] also tried to use CBTC (communication-based train control system) to realize unmanned rail transportation in underground mines. Zhao, C. [71] endeavored to use smart scrapers (LHD) as basic equipment in underground mines. Intelligent mines in China develop rapidly, and the development of intelligent mines also drives the development and progress of the backfill mining method. Qi Chong et al. [72,73] applied artificial intelligence to the backfill mining method (predicting the strength of the backfill slurry).

### 3.2. Key Technologies of Green Mine Construction in China

In order to solve the above mentioned in environment pollution, solid waste emissions, problems in the agricultural production, and the health of residents from the perspective of the mining technology, China endeavors to realize the harmonious development of resources development and environmental protection, reducing emissions of pollutants, and the damage to the environment, so as to eliminate or reduce the negative effects of economic, social, cultural, etc. Under this background and logic, China’s mining industry has put forward the development concept of green mining and promoted the construction of green mines [74].

Therefore, in this part, the paper mainly introduces the key technologies of green mine construction in China:

The Chinese government has clearly put forward in the process of constructing green mining, that mining waste rock, tailings, and other solid waste disposal rate must reach 100%, and standard sewage discharge rate must reach 100%.

In China, the difficulties in the construction of a green mine mainly include harmless disposal technology of solid wastes such as coal gangue and tailings, tail water purification, and recycling technology. The overall solution proposed by the Chinese government is

(1) To recycle most of the solid waste as filling aggregate and transport it to the mined-out area through pipelines, so as to eliminate hidden dangers in the mined-out area and prevent the surface collapse;
(2) A small amount of the remaining part can be used as a dry pile on the surface after dehydration or as building materials for secondary recycling, and the tailings pond can be canceled;
(3) Ecological treatment and reclamation of a dry storage yard to eliminate pollution sources. The concentrated or defiltered wastewater is recycled after purification or discharged up to standard.

Therefore, under the current economic and technological conditions in China, the construction of green mine mainly includes three key technologies: backfill (filling) mining method, dry heap of tailings, and wastewater recycling [74,75].

#### 3.2.1. Backfill Mining Method

The backfill method is the first method used in non-ferrous metal mines and precious metal mines, because it can maximize the recovery of underground mineral resources, protect the surface environment, and construction. In recent years as the backfill material is widely used in mines due to the advantages of the backfill process and equipment pipeline technology progress, reduced costs, and especially the country’s emphasis on safety and environmental protection. The reason are as follows:

(1) The goaf can be filled in time to effectively control ground pressure activities and avoid casualties caused by ground pressure disasters. There is no case of large-scale ground pressure disasters when backfill mining is adopted.
(2) Timely backfill of goaf can prevent movement and settlement of upper rock mass, and effectively protect the overlying coal seam from mining.
(3) Underground mineral resources can be recovered to the maximum extent. Compared to the open stoping method, the ore recovery is generally improved by 20~30%, dilution rate can be controlled to 8%. For example, at Gu Shan iron ore, using the backfill method instead of open stope method, the ore recovery rate rose from 60% to more than 90%, and the dilution rate is only 5%. The ore recovery rate of the Jinchuan nickel mine is 95% using backfill method.
(4) It can effectively deal with industrial solid waste and reduce the discharge of solid matter. Due to the large amount of backfill material, backfill not only reduces the discharge of solid matter, saves the cost of land acquisition and is a harmless treatment, it also more effectively reduces environmental pollution and opens an important method for the realization of green mines and mine surface environment treatment.

To this end, relevant national departments in China have issued a series of laws and regulations to encourage and guide the promotion of backfill method from the policy level [76].

At present, China has made rapid progress in green mining technology, especially in the development of the backfill mining method. Chinese researchers have made important contributions to the world. For example, in 2012, Ju, F. [77] planned to apply the backfill mining method to coal mining and designed a simulation program to test the feasibility of the backfill mining method in coal mining, which was a leap forward in China’s safe mining operations and green mine construction. In addition, in 2012, Deng, D. et al. [78] mainly applied ultra-high-water materials into the backfill mining method of a gold mine and explored the strength of backfill. Meanwhile, Li, S. et al. [79,80,81] studied the application of the Bayer processed red mud (BRM) to fill the goaf.

#### 3.2.2. Dry Heap Tailings (Dry Tailings Heap)

**Figure 5:**
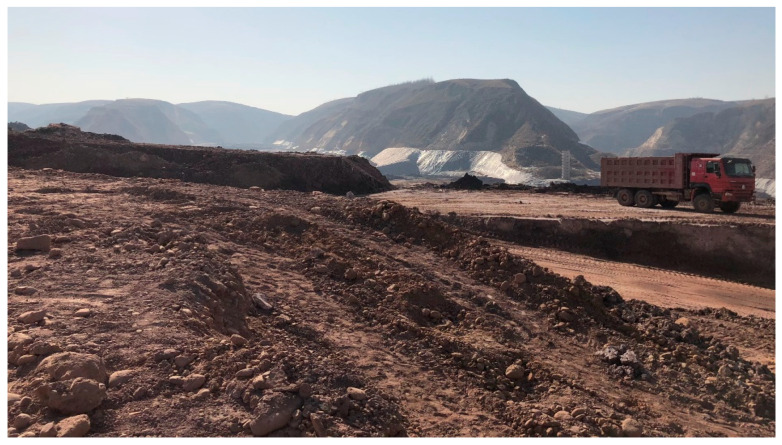
(Picture taken by authors.) Shanxi Huaxing aluminum Shentang ditch red mud dry stockpiling in China.

Dry heap tailings is a process that adopts filtration equipment to dehydrate tailings to a filter cake with a water content of less than 20%, and then transport it to a tailings stockpile by car or belt for dry stockpiling. The earliest dry tailings pile practice began in Australia in 1980 in the flat, red mud dry test pile disposal factory of an aluminum company. Then, with dry stacked technology’s rapid development, by the end of 2014 there were 463 tailings applying the technology. The alumina industry have all adopt the red mud dry storage process (see Figure 5). In 2010, China’s Ministry of Land and Resources officially issued policy documents requiring the full implementation of mineral resources planning, vigorously promoting tailings backfill and dry discharge technology, the development of green mining, the construction of green mines [82].

Compared with the traditional low-concentration directly discharged tailings ponds, the advantages of dry tailings heaps are as follows:

(1) The safety performance of the tailings pond is improved. The water content of the filter cake of the tailings after concentration and pressure filtration is low, and there is no water in the dry tailings yard. The stacking strength of the tailings after compaction is further improved, and the safety performance is greatly improved. The tailings cake is unsaturated, it is not easy to liquefy and has high shear strength, which greatly improves the seismic and flood prevention performance of tailings pond. Even if a dam break occurs, the dry tailings will not cause landslides, debris flow, or other disasters, and the damage degree is limited.
(2) Ecological and environmental pollution has been greatly reduced. The overflow water after tailing concentration is usually reused as mineral processing water, which greatly reduces the infiltration pollution of heavy metal ions and selected agents in the wastewater. Because there is no water in the storage, the dry storage yard can be built during reclamation;
(3) Reduced footprint and land acquisition costs. Due to the low water content of the tailings filter cake and the natural stockpiling which does not leak water, dry stockpiling has strong applicability to different topographic conditions, and can be safely stockpiled in canyons, or low-lying, flat land, or gentle slopes and other topographic conditions, thus greatly reducing the occupation area and land acquisition cost of tailings.
(4) The service life of the tailings pond is effectively extended. After a dry heap is used, the tailings accumulation density increases, and the total amount and service life increase greatly under the same storage capacity condition.
(5) Water savings. Dry heap tailings’ water return rate is more than 90%. Not only does this save precious water resources, especially in severe water shortage areas, but also realizes zero discharge of wastewater, which reduces the risk of environmental pollution;
(6) Recovery of valuable elements and recycling of mineral processing agents. The valuable elements and beneficiation agents in the wastewater can be effectively recovered and utilized because of the high water return rate of dry heap tailings.
(7) The cost of construction, operation, closure, and reclamation of the conventional tailings pond is reduced. The cost of construction, daily monitoring, maintenance, drainage, and infiltration treatment of traditional tailings ponds is as high as 5~10 RMB yuan/t, while the cost of a dry tailings pond is very low, only about half of that of traditional tailings pond.
(8) Strong adaptability to different regions, climate, and environment. The dry tailings heap has been successfully applied in rainy, arid areas, high seismic intensity and alpine areas, so the dry tailings heap has a wide application value.

#### 3.2.3. Wastewater Recycling

At present, China’s mining consumption of water resources, low recycling efficiency, heavy metal pollution, and other problems are very prominent, which not only further aggravates the local water shortage dilemma, but will also cause serious damage to local drinking water sources, crops, and ecological environments. Therefore, it is of vital importance and significance to adopt appropriate wastewater disposal technology to treat and comprehensively utilize mine wastewater for promoting the economic development of mining area and its region and the sustainable development of the whole mineral industry [83].

**Figure 6:**
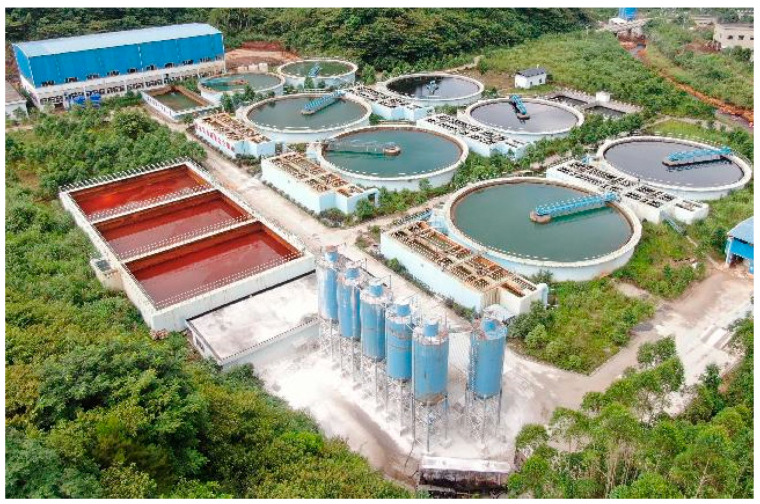
(Source from the Internet: https://www.sohu.com/a/419835808_120179917, accessed on 26 March 2022) Guangdong Dabaoshan Mining Co., Ltd. (Shaoguan, China). Mine sewage treatment system in China.

In addition to a small amount of domestic sewage, the main sources of mine sewage are mine gushing water and mineral processing tail water. Domestic sewage is the wastewater produced by the life of the mining area. It is small in scale, easy to treat, and has very mature integrated wastewater treatment equipment. Mine water is derived from the ore body during mining and prospecting. Fissure water, backfill exudation, and drilling water, have a general hardness and high salinity, and internally have tiny, suspended matter, such as dust and ash, fluoride, sulfide, such as inorganic salts. There is a need for special purification treatment to recycle or attain a discharging standard. Beneficiation tail water refers to the water contained in tailings discharged after the end of the beneficiation process. It generally contains a large number of beneficiation agents and heavy metal ions and often exceeds the standard of acid and alkali. It must be treated by special purification to be able to be recycled or reach the standard of discharge. While the mined-out area and surface dry backfill pile technology can effectively solve the mine main solid waste problem with a harmless disposal solution, there is a need to address wastewater. The overflow of the tailings concentrated water and filter backwater, can be treated by adding a flocculating agent to one or more paragraphs enrichment, initiating the flocculating sedimentation purification treatment process (see Figure 6), and thereby implement the direct reuse as the mineral water or obtain the discharge standard.

At present, the common mine sewage treatment processes and technologies in China are shown as the following:

(1) Coagulation and precipitation technology. Coagulation and precipitation technology is an important physical and chemical treatment method, usually using aluminum salt or iron salt as the coagulant. Evenly mixed sewage can complete purification treatment after precipitation and clarification. In recent years, due to the simple process and low cost, integrated coagulation and precipitation processing of sewage treatment equipment has been widely used, and the treated water can be directly discharged after filtration and disinfection.
(2) Microbial treatment technology. The technology uses the surface of the filler in the filter as a carrier, adsorption of organic matter flowing through the water, and then uses the oxidation of microorganisms on the surface of the biofilm to form a food chain composed of organic matter–bacteria–protozoa. The process is short, occupies a small area, and has high effluent quality, which is suitable for the reproduction of slow-growing microorganisms, such as nitrifying bacteria, and has strong ammonia nitrogen removal ability.
(3) Adsorption technology. At present, the most commonly used adsorption materials are activated carbon and diatomite, but activated carbon gradually loses its adsorption capacity with the extension of treatment time, so it needs to be replaced or regenerated activated carbon in time. Diatomite has a large number of orderly arranged micropores with strong adsorption capacity. It can absorb 1.5–4 times the weight of liquid and 1.1~1.5 times of oil, and the adsorption tower created also has a screening and depth effect, showing a good depth treatment effect.
(4) Reverse osmosis technology. This technology is a membrane separation technology driven by pressure. It has the advantages of no phase change, a simple process, small footprint, low energy consumption, and high pollutant removal rate. It has broad application prospects in coal mine wastewater treatment.
(5) Integrated membrane technology. By integrating ultrafiltration, microfiltration and reverse osmosis, the reverse osmosis membrane can greatly prolong the service life of the integrated membrane. Using ultrafiltration and microfiltration as the pretreatment process prior to reverse osmosis technology can ensure that the effluent water quality is at least above the tertiary water quality. This can greatly simplify the traditional sewage treatment pretreatment system.
(6) Continuous membrane filtration technology. This technology mostly uses low-cost hollow fiber, which can realize reverse flushing without a support layer, and has great application potential in the field of mine sewage treatment.

### 3.3. Reuse of Mining Waste

**Figure 7:**
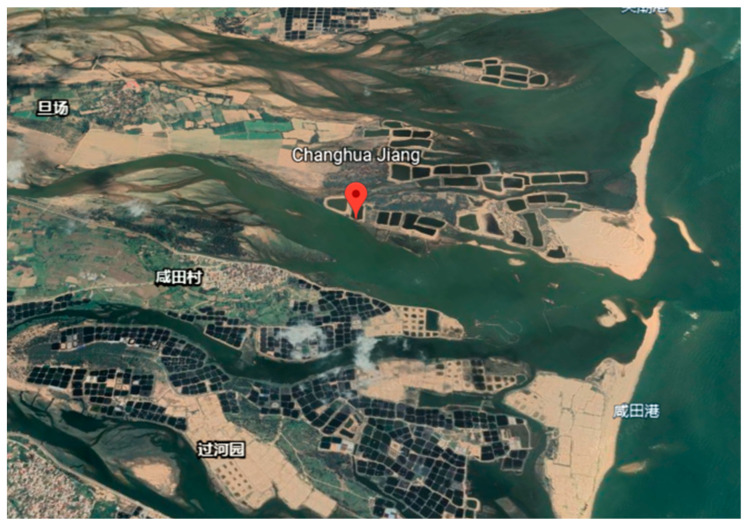
(Source from the Internet: https://earth.google.com/web/search/%E6%98%8C%E5%8C%96%E6%B1%9F/@19.30249965,108.6669444,2.13492146a,5315.58433093d,35y,71.39627759h,45t,0r/data=CigiJgokCcXyjL7sUjNAEdqMUQngQjNAGZRGx9K0LFtAIckptsh4J1tA, accessed on 26 March 2022) Satellite map of Hainan Changjiang Coast and Changhua River (Hainan, China).

At present, sand mining can cause serious environmental impacts, such as groundwater pollution and river pollution. For example:

A few years ago, the coastal sand mining activities in Changhua River (as shown in Figure 7) were very frequent, which had a very negative impact on the estuary in the Changhua River, the coastal sediments, and the coastal environment [84]: (1) After sand frequent mining sand in the downstream of Changhua River, the water level of Changhua River gradually became deeper. (2) Coastal mining has changed the dynamics of the Changhua River, resulting in the salinization of farmlands and harming local agriculture and crop growth. (3) Coastal mining leads to shoreline erosion, which affects the coastal environment and people’s lives.

Therefore, the use of mine waste in the preparation of concrete materials (especially in Road and Bridge construction) is also an important link in the development of green mine construction. Engineer Wanjun Jiang from the China Road and Bridge Corporation has carried out relevant studies.

In addition to the resource development strategy of Green Mine Construction proposed by China, the EU also proposed the Sustainable Development Goal #12 [85] which calls for a substantial reduction in waste generation through prevention, reduction, recycling, and reuse.

Anthropogenic resources are stocks which are designated as “wastes” and found in the anthroposphere (mineral resources, energy resources, soil resources, water resources, biological resources) [86]. Many people believe that anthropogenic resources provide or can provide useful benefits through the provision of secondary raw materials and energy used in the economic activity. The recycling and reuse of mining waste, in particular, indeed, would bring many positive benefits:

(1) Tailings and waste rocks from mining activities are often used as construction materials, which will reduce the cost of construction and bring positive economic benefits. As early as 2014, Oluwasola et al. [87] proposed that tailings had the potential to be used as building and construction materials;
(2) Mining waste, such as tailings, red mud, waste rocks, etc., can be used as backfill materials and backfill aggregates in the backfill mining method, which can save significant mining costs. At present, the application of mining waste to backfill has become a common practice in various mining enterprises in China [8,9,88];
(3) The main component of mining waste, such as tailings and coal gangue, is silica (SiO_2_), therefore, the mining wastes are often used to make inorganic products, such as magnetic sheath and glass, bringing good economic effect [89];
(4) Tailings [90] and red mud [91] can be used to produce fertilizers, and such waste reuse and recycling would undoubtedly have a positive impact on agricultural development.

According to relevant statistics, China’s current mining waste pile stock is more than 20 billion tons, which greatly limits the progress of China’s resource development strategy of Green Mine Construction. Meanwhile, mining waste also causes serious damage to the surrounding environment, which may even endanger the personal safety and health of local people. However, at the same time, mining waste, as a kind of anthropogenic resource, is also a potential secondary resource. If we can make full use of this kind of anthropogenic resource and take effective measures for mining waste recycling, mining waste could also become valuable.

## 4. Conclusions

(1) As a medium to guide readers to understand the development of China’s mining industry, this information article (Part I) systematically reviews the research progress and current situation of the Green Mine Construction in China, which plays a role in the future development of Green Mine Construction;
(2) This paper provides an extensive introduction to difficulties encountered in the development process of green mine construction, that is, groundwater pollution. Further, the paper describes the negative impact of mining activities on the environment, groundwater, farmland, and residents’ health, and highlights the necessity of mining pollution prevention and control in green mine construction;
(3) In addition, this paper extensively introduces the development process of “green” mines in China and introduces the three stages of the development of green mines in China through examples. At the same time, in the third chapter, the paper introduces some key technologies in green mine construction, such as the backfill mining method;
(4) Finally, the use of mining waste in building materials is a resource development strategy for resource reuse, and the paper notes that the use of mine waste in the preparation of building concrete materials (especially in road and bridge construction) is also an important link in the development of green mine construction.

We claim that the case report serves as a guide to starting a conversation, and we hope many more experts and scholars will be interested and engage in research in this field. At the same time, we also call on relevant researchers to actively invest in the research on green mining technologies, and promote the rapid development of Green Mine Construction, which will undoubtedly bring good news to people all over the world.

## Data Availability

Not applicable.
